# Effects of Initial Age and Severity on Cranial Remolding Orthotic Treatment for Infants with Deformational Plagiocephaly

**DOI:** 10.3390/jcm8081097

**Published:** 2019-07-24

**Authors:** Tiffany Graham, Beverley Adams-Huet, Nicole Gilbert, Kirsten Witthoff, Terran Gregory, Mary Walsh

**Affiliations:** 1 Health Care Sciences, Prosthetics-Orthotics Program, University of Texas Southwestern Medical Center, 6011 Harry Hines Blvd, Dallas, TX 75390-9091, USA; 2 Population and Data Sciences, University of Texas Southwestern Medical Center, 5323 Harry Hines Blvd, Dallas, TX 75390-8822, USA

**Keywords:** deformational plagiocephaly, plagiocephaly, cranial flattening, flat head syndrome, orthotic devices, treatment outcomes, cranial vault asymmetry (CVA), cranial vault asymmetry index (CVAI), cranial orthosis

## Abstract

The aim of this study is to review the effects of an infant’s presenting age and severity of deformation on cranial remolding orthotic (CRO) treatment outcomes for patients with deformational plagiocephaly. This study is a retrospective chart review of 499 infants with non-synostotic plagiocephaly who completed CRO treatment. Data collected included age at start of treatment, head shape measurements before and after treatment, total months of CRO treatment, and other factors such as presence/absence of prematurity. The infants were divided into subgroups according to age and severity at initiation of treatment and data for subgroups was analyzed to track the change in head shape over the course of treatment, review overall treatment duration, and discuss the rate of change of cranial deformation. Overall, treatment times tended to statistically increase with increasing initial severity and age. Posttreatment asymmetry measurements statistically trended to greater residual deformation in infants who began treatment in the older or more severe subcategories. This indicates that younger and less severe infants have shorter treatment durations and less residual cranial deformation after CRO treatment. Therefore, clinical consideration may need to be taken to treat infants at younger ages or prior to progression of the cranial deformity.

## 1. Introduction

Since the implementation of the “Back to Sleep” campaign by the American Academy of Pediatrics in the early 1990s [1,2,3,4,5,6,7,8], pediatricians in the United States have recommended infants sleep on their back, on a firm mattress, alone (no co-sleeping), and without items such as pillows or stuffed animals in the crib [9]. This initiative did result in a significant decrease in the incidence of Sudden Infant Death Syndrome (SIDS) [1,2,3]; however, some studies claim up to a six-fold increase in the diagnoses of deformational head shapes [4], with reports of up to 45% of 2-month-old infants having cranial deformation [5].

The term deformational plagiocephaly refers to a flattening of the skull due to the application of persistent external directional forces [4,6]. Some documented risk factors of deformational plagiocephaly include multiple gestation, prematurity, male sex, and the presence of congenital muscular torticollis (CMT) [1,10]. There is evidence that if left untreated, deformation of the cranium may become permanent [7].

Currently, a cranial remolding orthosis is a common treatment method within the United States and has been shown in several studies to be effective in reducing cranial asymmetry [1,2,11], although other treatment methods do exist [1,5,10]. A systematic review of the evidence published in 2016 by the Congress of Neurological Surgeons examined the most common treatment methods for deformational plagiocephaly: repositioning strategies, physical therapy, and cranial remolding orthoses (CROs) [10]. They found evidence to support the use of CROs for moderate to severe plagiocephaly and therefore recommended CRO use for these cases as well as infants who are diagnosed at an older age [10].

The cranial remolding orthoses described in this study were custom made to the infant’s head through the use of a 3D shape capture system, but can also be made by taking a plaster cast of the infant. The CRO is designed to gently direct skull growth by fitting snugly fit over bossed areas of the skull and by providing space in areas where skull growth is desired [6]. As such, it has been shown that the amount of correction an infant achieves is directly correlated to compliance with the wear schedule [7] and the amount of growth the infant achieves while in treatment [1,7].

Specific recommendations for the age at which a CRO should be initiated and certain treatment outcomes are still debated in the current literature [1,6,11]. For example, Seruya’s study found that correction rates decrease with increasing age of CRO initiation [11], while Freudlsperger’s study concludes that other interventions can be used prior to application of a CRO for infants with mild deformities, as the delay in treatment will not affect their correction rate [2]. Although the Congress of Neurological Surgeons does recommend CRO treatment in certain cases, they have not defined the cranial measurements or specific age at which an infant is indicated for a CRO [10]. This retrospective chart review was performed to assess evidence in a semi-controlled environment about specific outcomes of orthotic treatment of deformational plagiocephaly (treatment time and overall cranial correction) based on an infant’s presenting age and severity.

In defining key terms:

- Synostotic deformation is in reference to one or more prematurely fused cranial sutures in an infant.
- Plagiocephaly is the flattening of the skull to one side of the back of the head, with possible facial asymmetry and possible contralateral anterior flattening of the skull.
- Cranial Index (CI) is found by dividing the head’s width by its length, then multiplying by 100. Measurements are taken at the greater equator of the skull [3].
- Cranial Vault Asymmetry (CVA) is the diagonal difference (also known as oblique diagonal difference or transcranial difference). This is found by subtracting the larger cranial diagonal from the smaller cranial diagonal. Diagonals are taken from the lateral-anterior skull to the opposite side posterior skull at the level of the greater equator of the skull. These diagonals are taken 30 degrees clockwise and counterclockwise from the mid-sagittal line [12].
- Cranial Vault Asymmetry Index (CVAI) defined in this study is a modified version of the equation used by Loveday. It is the measurement of the CVA, indexed for the overall patient’s size. This is the absolute value of the difference in cranial diagonals (CVA), divided by the greater diagonal, then multiplied by 100. This allows for direct comparison between cranial deformities in infants with varying head sizes [4].

The CROs used in this study are STARbands manufactured by Orthomerica in Orlando, Florida, USA. These orthoses are all custom fabricated in a consistent and controlled manner, regulated by the Food and Drug Administration (FDA). The three offices included in this retrospective study reported that they trained their clinicians in the same manner and used the same methods to evaluate and measure their patients. The follow-up schedule was more frequent at the beginning of treatment, decreasing in frequency toward the end of treatment (generally every 1–4 weeks). During follow-up visits, compliance was discussed and encouraged (the recommended 23 hour per day wear schedule was emphasized), growth progress examined, and the CRO was adjusted to continue to direct growth of the skull.

During the time the patients were undergoing treatment, the STARscanner was used to create a 3-dimensional computerized image of the subjects in the Yeti Shape Builder program. The STARscanner is a laser data acquisition system which collects the subject’s head shape in under two seconds. After being scanned, the image from Yeti Shape Builder was uploaded into the Cranial Comparison Utility (CCU) program which produces a STARscan report. This STARscan report includes measurements of the patient’s head, including CVAI, and is recorded in the patient’s records. The STARscanner hardware, Yeti Shape Builder software, and CCU software are manufactured by Vorum Research Corporation in Vancouver, Canada. Scans of the infants were taken at 4–8-week intervals throughout treatment. The scan measurements used in this study were taken from the scan used for CRO fabrication and the scan used to discharge the patient from treatment. The STARscanner has been proven to be a reliable measurement tool for cranial shape capture [13].

## 2. Materials and Methods

This retrospective chart review was conducted in accordance with the Declaration of Helsinki and the protocol was approved with a waiver of consent by the University of Texas Southwestern Medical Center’s Institutional Review Board (IRB Number: STU 032016-077). A data transfer agreement was granted through Level 4 Prosthetics & Orthotics and UT Southwestern (Contract Number: OTD-110814). The charts reviewed were infants who underwent CRO treatment in one of the three Texas offices of Level 4 Prosthetics & Orthotics (one office in Addison, Texas and two in San Antonio, Texas).

To be included in the study, the subjects must have been diagnosed with deformational plagiocephaly by a pediatrician, neurosurgeon, craniofacial surgeon, or plastic surgeon and referred to Level 4 Prosthetic and Orthotic clinics for treatment. Also, the subjects must have initiated treatment between 3 and 18 months of age and completed treatment between January 2013 and June 2017. Subjects were excluded if they had any other positional head deformities (such as brachycephaly, scaphocephaly, or asymmetrical brachycephaly/scaphocephaly). For the purpose of this study, infants with isolated plagiocephaly were defined as having a CI < 90% and a CVAI ≥ 3.0. Subjects who had synostotic head shapes or significant comorbidities other than prematurity and torticollis were excluded from the study as these factors may affect growth rates. Subjects with torticollis or subjects born prematurely (defined in this study to infants born prior to 38 weeks gestation) were included. Premature subjects were age-adjusted through calculating the postpartum age minus the number of weeks of prematurity, then rounding to the nearest whole month. (i.e., a 4-month-old subject born at 36 weeks gestation was categorized as a 3-month-old subject and noted to be positive for prematurity). In a previous analysis by this cohort, this treatment age correction was shown to produce results which were not statistically different from other full-term infants of the same treatment age [14]. Subjects who dropped out of treatment, subjects who did not complete treatment despite practitioner recommendation, or subjects who were lost to follow-up were excluded. Patient chart information gathered included corrected age at start and end of CRO treatment, presence or absence of prematurity, CVAI at the start and end of treatment, and treatment duration. Treatment initiation age was calculated as the nearest whole month postpartum, corrected for prematurity. The cranial deformation was measured using the CVAI. The change in CVAI was calculated as the final CVAI minus the initial CVAI. Treatment time was rounded to the nearest whole month of orthotic treatment.

The included subjects were divided into subgroups according to their presenting severity and age at the onset of treatment. Severity categories were based on the Children’s Healthcare of Atlanta Plagiocephaly Severity Scale [15]. For each subgroup, the mean treatment duration and standard deviation were calculated. The Jonckheere–Terpstra test was performed to test for trends across the age and severity categories. Data analysis was performed using Microsoft Excel 2013, SAS 9.4, and SPSS 25. For statistical analysis, the significance was set to *p* < 0.05.

## 3. Results

In total, 2423 charts were reviewed and 499 included. The exclusion breakdown is as follows: 1402 had non-plagiocephalic deformational head shapes, 201 had synostotic head shapes, 73 had significant comorbidities (other than prematurity and torticollis), 204 dropped out, 27 were non-compliant, 8 had incomplete data in their charts, 4 had treatment extend beyond June 2017, 3 moved out of state during the course of treatment, 1 discharged early, and 1 did not meet the age requirements for the study.

The 499 included subjects spanned a variety of initial treatment ages and severities. Of these subjects, 300 subjects were found to be positive for torticollis and 142 were born prior to 38 weeks gestation. There was an overlap of 93 patients who were positive for both torticollis and prematurity. The premature patients were assigned to groups according to their corrected age as previously described (postpartum age minus the number of weeks of prematurity, rounded to the nearest whole month). All subjects began treatment at a corrected age between 2 and 17 months and had CVAI measurements spanning from 3.1 to 16.1. All subjects ended treatment at a corrected age between 5 months and 21 months and had final CVAI measurements between 0.1 and 10.1.

The distribution of subjects for each age and severity subgroup is shown in Table 1. Treatment initiation ages were grouped as shown in Table 2. The initial severity classification corresponded to the Children’s Healthcare of Atlanta Plagiocephaly Severity Scale [15], where Mild had a CVAI < 6.25, Moderate had a CVAI of 6.25–8.75, Severe had a CVAI of 8.75–11, and Very Severe had a CVAI > 11. The age classification is as follows: Very Early infants began treatment before 4 months of age, Early infants began treatment at 4–5 months of age, Mid infants began treatment at 6–7 months of age, Late infants began treatment at 8–10 months of age and Very Late infants began treatment at 11–18 months of age.

These groups were then split into subgroups based on age and severity, which yielded 20 subcategories for age and severity combinations. Based on the collected data, the Very Late–Very Severe subgroup did not have any data to report as no subjects fell into this category. The mean treatment time for each subcategory was calculated and the standard deviation applied. The division of subjects and respective mean treatment times and results of the Jonckheere–Terpstra test are shown in Table 3.

Treatment duration statistically trended to increase with severity for each age group with the exception of the Very Early age group. Similarly, the statistical trend for treatment duration significantly increased with age within each isolated severity category except the Mild severity.

**Figure 1:**
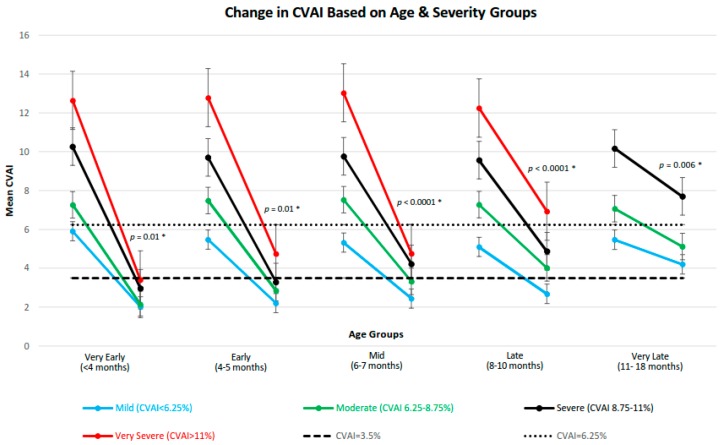
Mean change in CVAI with standard of deviations in CVAI before and after CRO treatment for each subcategory (*n* = 499). *p*-values are from Jonckheere–Terpstra test for trend which shows the trend of final CVAI within severity categories as age increases. CVAI: Cranial Vault Asymmetry Index, CRO: Cranial Remolding Orthosis. * indicates significance (*p* < 0.05).

The change in the CVAI over the course of treatment was assessed for each subgroup (Figure 1). The final CVAI measurement has an increasing trend as initial severity worsens across all age categories, which represents increasing post-treatment cranial deformation. This was statistically significant in each age group as shown by the Jonckheere–Terpstra test for trend *p*-values in Figure 1.

The same trend test was performed across the age groups to examine if increasing initial age has an effect on final CVAI within each severity category. This test yielded *p* = 0.006 for Mild, *p* < 0.0001 for Moderate, *p* < 0.0001 for Severe, and *p* = 0.0009 for Very Severe treatment groups, indicating that an increase in age is associated with an increase the final CVAI measurements.

The overall change in CVAI following treatment was also associated with severity; trend tests were significant in all age groups except in the Very Late age group with *p*-trend values of *p* < 0.0001 and *p* = 0.33 respectively. This indicates that patients with larger CVAI differences over the course of treatment generally had more severe starting presentations, with the exception of the very late age group.

Supplementary data is provided in Table S1 and Figure S1. Table S1 shows the descriptive statistics of each age and severity sub-group’s starting and ending CVAI measurements. In Figure S1, all data from these age and severity subgroups is plotted with their final CVAI measurement based on their initial CVAI grouping. Trend lines were added to show how increasing starting CVAI generally increases the ending CVAI and seems to have different effects for each age group (older groups generally have higher ending CVAI measurements).

## 4. Discussion

**Figure 2:**
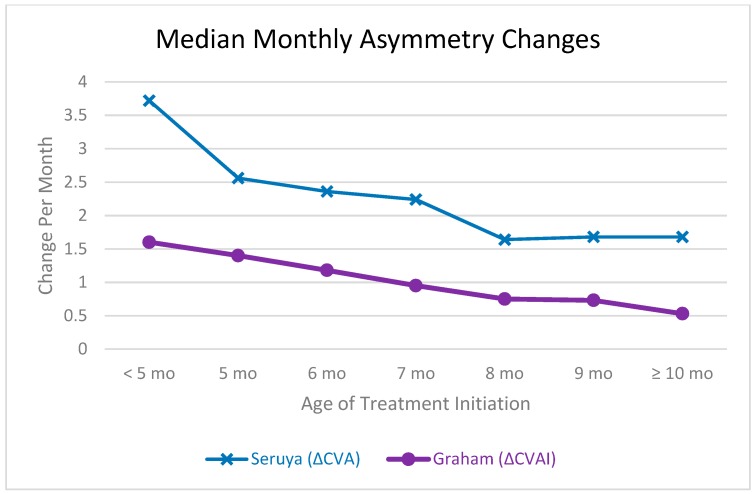
Study comparison of median monthly asymmetry changes in CVA or CVAI based on infant starting age (Seruya *n* = 346, Graham *n* = 498).

The optimal age to begin orthotic treatment has been suggested by multiple studies, all with various recommendations [1,2,3,11,16]. In order to better discuss our study results, a comparison to other studies should be made. Figure 2 is adapted from the 2013 manuscript by Seruya et al. [11] and can somewhat compare our results, with limitations. This figure can be used to show that the correction rate of infants substantially decreases with the age of initiation. Our previous analysis also demonstrated that the monthly mean rate of change for infants with deformational plagiocephaly decreases with increasing age [14]. Trends found by Seruya et al. are similar, although a direct comparison cannot be drawn as our study used CVAI as the asymmetry measurement, while Seruya used CVA [11]. Although the CVA and CVAI are related, CVAI takes into account the percentage of deformity of the skull as the patient’s head circumference increases (by dividing by the greater of the cranial diagonal measurements). Therefore, if the CVA remains constant, CVAI will continue to decrease as a patient grows. That being said, the rate of CVAI change is still expected to decrease over time if CVA is constant because the growth of the skull slows with age. There are other limitations for a direct comparison as Seruya’s article does not mention if gestational duration was used, so it is possible that a direct correlation of age is not appropriate. Also, Seruya et al. included patients up to 15 months of age, but it was not clear if 15 months was the starting age or the finishing age of treatment. Similarly, the first age group were infants who began treatment at less than 20 weeks of age, but no lower limit was defined. In the numbers reported in Figure 2, we limited the comparison to having the oldest patient starting treatment at 15 months (who consequently ended treatment at 21 months) in an attempt to more accurately compare the studies [11]. Also, median measurements can be strongly influenced by the demographics of the data set. That being said, trends can be demonstrated in Figure 2.

**Figure 3:**
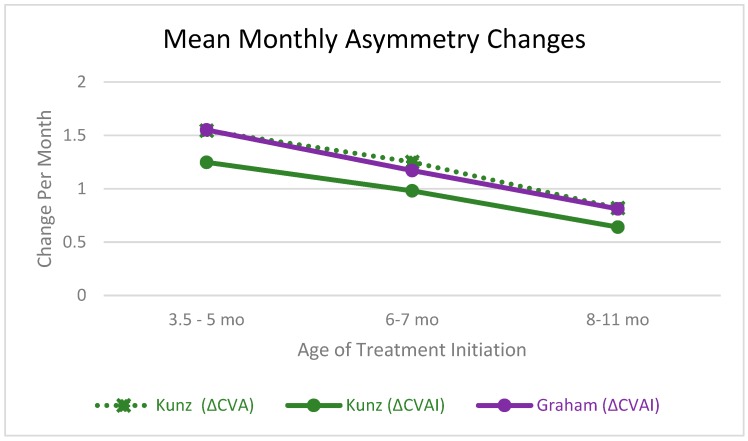
Study comparison of mean monthly asymmetry changes based on infant starting age (Kunz *n* = 144, Graham *n* = 473).

To visualize the relationship between the rate of change in CVA versus the rate of change in CVAI, the data from Kunz, et al. has been adapted in Figure 3 to show the rate of change of each variable in the age subgroups examined by that cohort [17]. In this graph, Kunz reported on 144 infants. To make a similar comparison based on starting age, we report on 473 patients. Kunz limited gestational age to a minimum of 37 weeks [17], so the age comparison should be similar since we corrected for prematurity.

The data collected in this study supports the results of the previous studies in that the overall correction for deformational head shape treatment with an orthosis is related to the age of initiation of CRO treatment [7,11,16,17], and treatment duration is shorter with younger infants [3,17]. Our study also supports the findings of Kunz and Seruya in that younger infants have greater correction rates [11,17]. We expand the examination of Kunz and Seruya by including a larger number of infants in this retrospective chart analysis as well as expanding the recorded age groups to anywhere between 3 and 18 months of age at the start of treatment. Previous studies report a difference in treatment outcomes for infants aged younger than 6 months or older than 6 months at the initiation of treatment [2,16]. In our analysis, treatment groups were divided into more narrow age and severity subsets than many previous studies to examine if these smaller treatment subgroups have statistical differences. Although treatment duration was not found to be statistically different for the Mild-Very Early group than the other groups, the mean final CVAI of the Mild-Very Early group was lower than in older or more severe groups, which indicates that this group did have overall better correction of the deformational head shape.

It is possible that the trend of greater and faster correction in younger age groups is due to the changing rate of circumferential cranial growth during normal infant development. According to a recent examination of normal calvarial growth between 4 and 10 months of life (taken at 2 months intervals), the greatest increase in neurocranial volume occurred in the 4–6 months age range, which is the youngest range recorded by the study [18]. The Center for Disease Control’s well published normal cranial circumferential growth chart also shows the rate of growth is faster for younger infants [19].

In general, the treating orthotists for this study were trained to recommend for CRO treatment to continue until the head shape is normalized or the infant is no longer growing a clinically significant amount (i.e., no longer seeming to change in overall circumference or asymmetry measurements). For this reason, our finding of more severe infants achieving greater correction is not surprising, especially considering that longer treatment times were found in these severity groups. What is surprising, is that this CVAI difference was not significant in the Very Late age group, which indicates that infants who are treated starting at 11 months of age are less likely to have additional correction, even with an increase in treatment duration. Some studies have suggested that infants who initiate treatment after 12 months of age have poor treatment outcomes, including Mortenson, et al. [6]. Steinberg found that infants who began treatment after 12 months of age were 3.08 times more likely to fail orthotic treatment than 3- to 6-month-old infants [1]. Future studies of older infants should investigate if there is an age at which cranial correction halts.

Another important note is that the treating orthotists did not necessarily terminate CRO treatment immediately when the cranial shape was corrected. Specifically, the orthotists recommended waiting to discontinue treatment until the infant was either an active sleeper (moving around at night), tummy sleeper (which keeps pressure off the back of the skull), or sitting independently. The clinicians reported that in general, this recommendation only affected the treatment duration of the Very Early treatment group, which may not have achieved these developmental milestones by the time the head shape was corrected. This could be the reason that statistical trends were not found in the Very Early age group for treatment duration with increasing severity.

Limitations of this study include the use of only one company’s data, one brand of cranial remolding orthosis, and the inclusion of only isolated deformational plagiocephaly cases. Conclusions drawn from this study should not be applied to cases of asymmetrical brachycephaly, brachycephaly, or infants with comorbidities which may affect growth rates. Considering this study was a retrospective chart review among infants treated with a CRO, no untreated control group was able to be included. The Congress of Neurological Surgeons found “a fairly substantive body of non–randomized evidence that demonstrates more significant and faster improvement of cranial shape in infants with positional plagiocphaly treated with a helmet as compared to conservative therapy”; however, further studies are needed to assess the natural course of untreated cranial deformation [20].

Further studies should be done to examine CRO treatment outcome measurements at different patient care centers, with different orthoses manufacturers, and with different treating orthotists to see if these results are robust across the industry. Confounding variables to this study are patient compliance, uneven distribution of ages and severities, and the variability of individual growth rates. This study could be enhanced by increasing the number of included subjects, particularly subjects in older and more severe treatment subgroups.

## 5. Conclusions

Our study found statistical differences in the final CVAI measurement of all of the age groups as well as differences between the severity groups within all of the age groups. Older infants or infants with more severe initial cranial deformation had significantly worse posttreatment residual cranial deformation. Treatment times also tended to increase with increasing severity or initial age. Younger and less severe infants tend to have shorter treatment durations and less residual cranial deformation after CRO treatment. Therefore, clinical consideration may need to be taken to treat infants at younger ages or prior to progression of the cranial deformity. Of note, in the United States, the Food and Drug Administration regulates the treatment age to be between 3 and 18 months [21], so treatment outside of this age range has not been examined.

## Figures and Tables

**Table 1:** Division of subjects within severity categories (*n* = 499).

**CVAI**	<6.25	6.25–8.75	8.75–11	>11
**Classification**	Mild	Moderate	Severe	Very Severe
**Number of Subjects**	129	214	110	46

CVAI: Cranial Vault Asymmetry Index.

**Table 2:** Division of subjects based on treatment initiation age, corrected for prematurity (*n* = 499).

**Initial Age**	<4 Months	4–5 Months	6–7 Months	8–10 Months	≥11 Months
**Classification**	Very Early	Early	Mid	Late	Very Late
**Number of Subjects**	18	214	166	88	13

**Table 3:** Mean treatment durations ± standard deviations for each age and severity subgroup (*n* = 499).

Mean Treatment Duration (Months)							
		Age of CRO Initiation					
		Very Early	Early	Mid	Late	Very Late	*p*-Trend for Severity
**Initial Severity**	Mild	2.33 ± 0.58	3.26 ± 1.07	3.32 ± 1.19	3.56 ± 1.31	2.67 ± 0.58	0.27
		*n* = 3	*n* = 43	*n* = 53	*n* = 27	*n* = 3	
	Moderate	3.2 ± 0.58	3.28 ± 0.97	3.74 ± 1.03	4.14 ± 1.60	5.14 ± 1.46	<0.0001 *
		*n* = 5	*n* = 97	*n* = 63	*n* = 42	*n* = 7	
	Severe	4 ± 0	3.78 ± 1.14	5.05 ± 2.07	6.23 ± 1.96	5.67 ± 2.31	<0.0001 *
		*n* = 3	*n* = 50	*n* = 41	*n* = 13	*n* = 3	
	Very Severe	3.85 ± 1.86	5.08 ± 2.19	6 ± 2.50	7.67 ± 1.37		0.0003 *
		*n* = 7	*n* = 24	*n* = 9	*n* = 6	*n* = 0	
	*p*-Trend for Age	0.15	<0.0001 *	<0.0001 *	<0.0001 *	0.02 *	

* indicates significance (*p* < 0.05). CRO: Cranial Remolding Orthosis.

## Supplementary Materials

The following are available online at https://www.mdpi.com/2077-0383/8/8/1097/s1, Table S1. Descriptive statistics of the Age and Severity subgroups. Figure S1. Raw Data Plot showing how increasing severity groups affect the final CVAI, subdivided by initial age groups.
