# Supplementary material for: Effects of Initial Age and Severity on Cranial Remolding Orthotic Treatment for Infants with Deformational Plagiocephaly
Source: J Clin Med. 2019 Jul 24;8(8):1097. doi: 10.3390/jcm8081097 (PMC6723372; doi:10.3390/jcm8081097)
Supplement: Supplementary file 1 [file jcm-08-01097-s001.pdf]

Table S1. Descriptive statistics of the Age and Severity subgroups.

AgeGrp_new	SeverityClass	N Obs	Variable	N	Mean	Std Dev	Median	10th Pctl	25th Pctl	75th Pctl	90th Pctl	Minimum	Maximum
1VeryEarly	Mild	3	CVAIstart	3	5.9000	0.3606	6.0000	5.5000	5.5000	6.2000	6.2000	5.5000	6.2000
			CVAIend	3	2.0333	0.6429	2.3000	1.3000	1.3000	2.5000	2.5000	1.3000	2.5000
	Moderate	5	CVAIstart	5	7.2600	0.9476	6.8000	6.4000	6.6000	7.9000	8.6000	6.4000	8.6000
			CVAIend	5	2.1400	0.6189	2.3000	1.4000	1.7000	2.3000	3.0000	1.4000	3.0000
	Severe	3	CVAIstart	3	10.267	0.4163	10.400	9.8000	9.8000	10.600	10.600	9.8000	10.600
			CVAIend	3	2.9667	0.5774	3.3000	2.3000	2.3000	3.3000	3.3000	2.3000	3.3000
	Very severe	7	CVAIstart	7	12.643	1.4943	12.600	11.100	11.100	13.100	15.500	11.100	15.500
			CVAIend	7	3.4000	1.2570	3.1000	1.6000	2.5000	4.8000	4.9000	1.6000	4.9000
2Early	Mild	43	CVAIstart	43	5.4791	0.6606	5.6000	4.6000	5.1000	6.0000	6.2000	3.1000	6.2000
			CVAIend	43	2.2070	1.0736	1.9000	1.0000	1.5000	3.4000	3.7000	0.1000	4.3000
	Moderate	97	CVAIstart	97	7.4907	0.7106	7.4000	6.5000	7.0000	8.2000	8.5000	6.3000	8.7000
			CVAIend	97	2.8505	0.9205	3.0000	1.7000	2.2000	3.6000	3.9000	0.6000	5.5000
	Severe	50	CVAIstart	50	9.7000	0.5425	9.6000	9.0000	9.2000	10.100	10.450	8.8000	11.000
			CVAIend	50	3.2920	0.9245	3.4500	1.9500	2.8000	3.8000	4.2500	0.8000	5.8000
	Very severe	24	CVAIstart	24	12.783	1.4782	12.500	11.200	11.500	13.450	15.300	11.100	16.100
			CVAIend	24	4.7375	1.2645	4.7000	3.3000	3.8500	5.7500	6.3000	2.6000	7.5000
3Mid	Mild	53	CVAIstart	53	5.3170	0.7010	5.5000	4.4000	5.0000	5.8000	6.0000	3.5000	6.2000
			CVAIend	53	2.4434	0.9864	2.5000	1.3000	1.8000	3.3000	3.6000	0.2000	4.8000
	Moderate	63	CVAIstart	63	7.5238	0.6453	7.5000	6.7000	7.0000	8.1000	8.3000	6.3000	8.7000
			CVAIend	63	3.3254	0.9086	3.5000	2.3000	2.8000	3.8000	4.2000	0.5000	6.1000
	Severe	41	CVAIstart	41	9.7634	0.5748	9.7000	9.1000	9.2000	10.300	10.400	8.8000	10.900
			CVAIend	41	4.2244	1.0039	3.9000	3.3000	3.6000	4.9000	5.7000	2.4000	6.7000
	Very severe	9	CVAIstart	9	13.033	1.6591	12.900	11.100	11.800	13.700	16.100	11.100	16.100
			CVAIend	9	4.7556	1.7365	5.3000	2.4000	3.4000	5.4000	7.3000	2.4000	7.3000
4Late	Mild	27	CVAIstart	27	5.0963	0.8239	5.1000	3.6000	4.4000	5.7000	6.1000	3.3000	6.2000
			CVAIend	27	2.6815	1.0583	2.8000	1.3000	2.1000	3.6000	4.0000	0.2000	4.5000
	Moderate	42	CVAIstart	42	7.2786	0.7751	7.1000	6.3000	6.7000	8.0000	8.4000	6.3000	8.7000
			CVAIend	42	4.0238	1.1774	3.7500	2.7000	3.2000	4.7000	6.2000	2.3000	6.6000
	Severe	13	CVAIstart	13	9.5615	0.6345	9.4000	9.0000	9.0000	9.8000	10.200	8.8000	11.000
			CVAIend	13	4.8692	1.5107	5.2000	2.6000	3.8000	5.5000	6.7000	2.2000	7.5000
	Very severe	6	CVAIstart	6	12.250	1.1292	11.650	11.300	11.600	13.200	14.100	11.300	14.100
			CVAIend	6	6.9333	0.6121	6.9000	6.1000	6.6000	7.3000	7.8000	6.1000	7.8000
5VeryLate	Mild	3	CVAIstart	3	5.4667	0.6807	5.7000	4.7000	4.7000	6.0000	6.0000	4.7000	6.0000
			CVAIend	3	4.2000	0.7550	4.3000	3.4000	3.4000	4.9000	4.9000	3.4000	4.9000
	Moderate	7	CVAIstart	7	7.0714	0.7455	7.0000	6.3000	6.4000	7.6000	8.4000	6.3000	8.4000
			CVAIend	7	5.1143	1.2747	5.2000	3.5000	3.5000	6.2000	6.6000	3.5000	6.6000
	Severe	3	CVAIstart	3	10.167	0.5508	9.9000	9.8000	9.8000	10.800	10.800	9.8000	10.800
			CVAIend	3	7.7000	2.0881	6.7000	6.3000	6.3000	10.100	10.100	6.3000	10.100

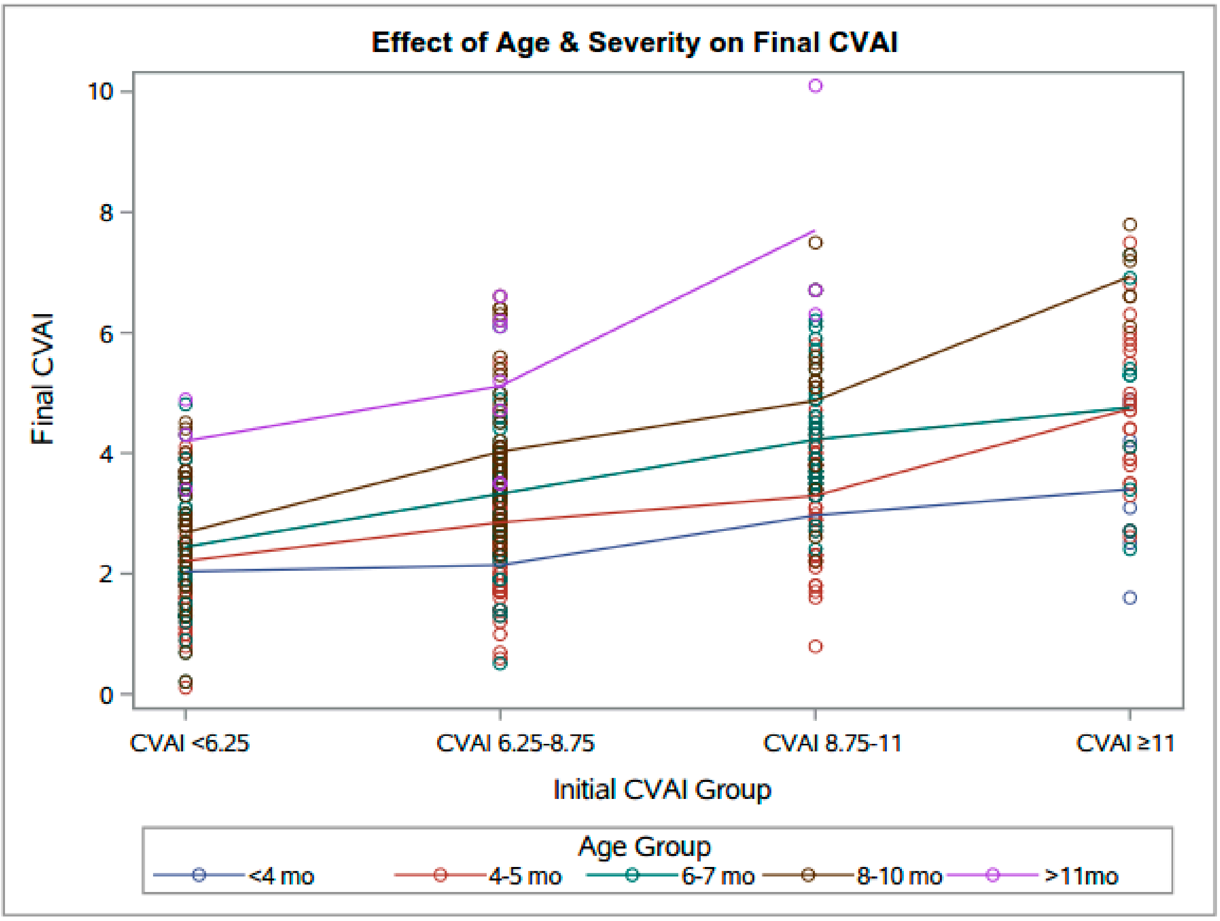

Figure S1. Raw Data Plot showing how increasing severity groups affect the final CVAI, subdivided by initial age groups.
